# BC1 RNA motifs required for dendritic transport *in vivo*

**DOI:** 10.1038/srep28300

**Published:** 2016-06-28

**Authors:** Thomas Robeck, Boris V. Skryabin, Timofey S. Rozhdestvensky, Anastasiya B. Skryabin, Jürgen Brosius

**Affiliations:** 1 Institute of Experimental Pathology, Center for Molecular Biology of Inflammation (ZMBE), University of Münster, Von-Esmarch-Str. 56, D-48149, Münster, Germany; 2 Department of Medicine (TRAM), University of Münster, Von-Esmarch-Str. 56, D-48149 Münster, Germany; 3 Institute of Evolutionary and Medical Genomics, Brandenburg Medical School (MHB), D-16816 Neuruppin, Germany

## Abstract

BC1 RNA is a small brain specific non-protein coding RNA. It is transported from the cell body into dendrites where it is involved in the fine-tuning translational control. Due to its compactness and established secondary structure, BC1 RNA is an ideal model for investigating the motifs necessary for dendritic localization. Previously, microinjection of *in vitro* transcribed BC1 RNA mutants into the soma of cultured primary neurons suggested the importance of RNA motifs for dendritic targeting. These *ex vivo* experiments identified a single bulged nucleotide (U_22_) and a putative K-turn (GA motif) structure required for dendritic localization or distal transport, respectively. We generated six transgenic mouse lines (three founders each) containing neuronally expressing BC1 RNA variants on a BC1 RNA knockout mouse background. In contrast to *ex vivo* data, we did not find indications of reduction or abolition of dendritic BC1 RNA localization in the mutants devoid of the GA motif or the bulged nucleotide. We confirmed the *ex vivo* data, which showed that the triloop terminal sequence had no consequence on dendritic transport. Interestingly, changing the triloop supporting structure completely abolished dendritic localization of BC1 RNA. We propose a novel RNA motif important for dendritic transport *in vivo*.

Over the past 15 years, numerous non-protein coding RNAs (RNAs) were identified in all kingdoms of life^1^. With the exception of messenger RNAs (mRNAs), these macromolecules do not encode proteins but are involved in the regulation of a remarkably broad spectrum of cellular processes usually in complex with proteins^2^. RNAs were shown to play important roles in normal cellular/organism development and physiology; their loss, mutation or dysregulation of expression was linked to various diseases^3^,^4^,^5^,^6^,^7^,^8^. Presently, there is an explosion of studies employing micro RNAs (miRNAs) as diagnostic tools, as well as recognizing the prominent roles they play in disease. A 2002 PubMed search for “miRNA and disease” generated only five hits; a current search reveals over 11,400 entries. Even evolutionarily young RNAs, such as the small brain cytoplasmic (BC1) RNA^9^,^10^ can have an impact on an organism when depleted: A BC1 RNA gene knockout in mice leads to a reduction in exploratory behavior and increased anxiety^11^,^12^. More recent reports suggests that the absence of BC1 RNA dependent translational control results in neuronal hyper-excitability and a propensity for epileptogenic responses^13^. BC1 RNA forms RNP particles^14^,^15^,^16^,^17^,^18^. The ~150 nucleotides of BC1 RNA can be divided into a tripartite structure^10^,^19^,^20^: The 5′-domain (or ID-region) exhibits a stem loop structure that was derived from a retroposed tRNA^Ala^. The central domain features an unstructured Adenosine-rich region as well as a short 3′ stem loop structure^20^. In most rodents, there is a single active gene that is transcribed by RNA polymerase III^21^. Its transcript is a source gene for a type of short interspersed repeat element (SINEs) termed ID elements^22^. The presumed functional analog of BC1 RNA in primates is BC200 RNA^23^. Both small RNAs are specifically expressed in neurons with low levels in testis and are transported from the cell body far into the dendrites^24^. BC RNAs are mediators of translational control and are involved in the local regulation of protein synthesis in the synaptodendritic compartment that underlies neuronal plasticity^25^,^26^,^27^,^28^,^29^. Despite the biological significance of RNA transport into dendrites, knowledge concerning the structural determinants necessary for this transport remains spotty^30^. In particular, discrepancies between *ex vivo* and *in vivo* experiments make it necessary to probe structure-function relationships in intact organisms - in our case, transgenic mice^31^.

In order to study potential RNA structures and/or motifs responsible for BC1 RNA transport *in vivo* we have generated a series of BC1 RNA variants and expressed them in transgenic mice. Our previously reported BC1−/− knockout mouse line was chosen as recipient background^11^. We examined the brains of the various mice by *in situ* hybridization with emphasis on the dendritic fields of the hippocampi. Once more, the *in vivo* results deviated to some degree from the previous *ex vivo* data^18^.

## Results

### Transgenes and RNA expression

We generated six transgenic mouse lines (Tg^BC1_UCU^, Tg^BC1_AUC^, Tg^BC1_AG^, Tg^BC1_GAU^, Tg^BC1_A^ and Tg^BC1^), which expressed mutant rat BC1 RNA as well as the rat BC1 wild-type molecule. Rat and mouse BC1 RNA are virtually identical. The genetic background for all transgenic lines was FVB/N lacking the BC1 RNA gene (BC1−/−)^11^. We generated at least three founders for each line and examined them via *in situ* hybridization.

**Figure 1:**
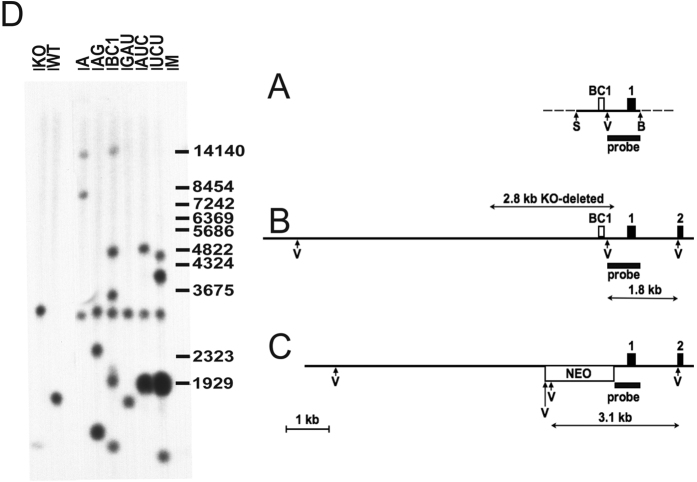
Southern blot analysis of BC1 transgenic mice. (**A**) Rat 1.4 kb genomic DNA fragment between *Sac*I (S) and *Bam*HI (B) restriction sites was used as basis for transgenes. Other *Sac*I and *Bam*HI sites are not shown; *Eco*RV (V) sites are indicated on all alleles. The BC1 RNA gene and the 5′ exon of the *Oraov1* gene are shown as open or black rectangles above the line, respectively. The DNA probe used in all Southern blots originated from the rat genome and spanned 801 nt between the indicated *Eco*RV and *Bam*HI sites. (**B**) Wild-type mouse genomic locus where the first two exons of the *Oraov1* gene are indicated as black rectangles and numbered accordingly. The 2.8 kb region deleted in BC1−/− is indicated by an arrow above the map. The 1.8 kb *Eco*RV fragment identified by the probe is shown by an arrow below the map. (**C**) Mouse genomic locus after replacing the BC1 RNA gene region with a neomycin resistance cassette (open rectangle under the line, marked NEO). Intronic and intergenic regions are shown as lines. The 3.1 kb *Eco*RV fragment identified by the probe is marked by an arrow. Note that, due to the deletion, the target sequence of the probe is only 595 nt instead of 801 nt on this allele. (**D**) Southern blot of *Eco*RV digested tail DNA samples of various transgenic, wild-type and BC1−/− mice. KO indicates FVB/N BC1−/− mice, which served as the recipients for the transgenes. FVB/N are the corresponding wild-type mice. A, AG, BC1, GAU, AUC, UCU are abbreviations of the various transgenic mice. A denotes Tg^BC1_A^ that harbored an insertion that removed the bulge U_22_; AG (Tg^BC1_AG^) alters the penultimate base-pair near the terminal loop, BC1 (Tg^BC1^) harbored the rat BC1 wild-type gene, GAU (Tg^BC1_GAU^) altered the sequence of the terminal triloop, and AUC (Tg^BC1_AUC^) and UCU (Tg^BC1_UCU^) affected the GA motif. The faint fast migrating band in the KO panel might be a contamination with the probe fragment.

**Figure 2:**
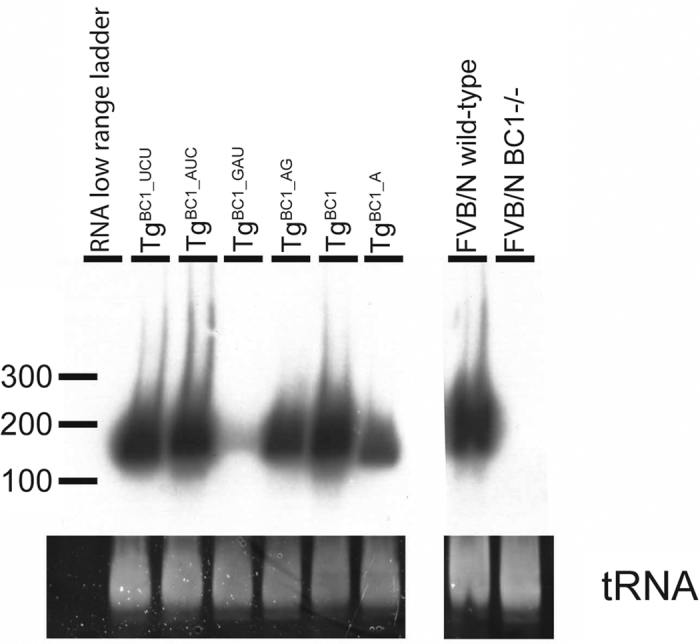
Northern-blot analysis of total brain RNA from BC1 transgenic mice. Northern blot autoradiographs of total brain RNA from BC1 transgenic mice, FVB/N wild-type and FVB/N BC1−/− mouse brains are shown in the upper panels (exposure time: ~20 h). Ethidium bromide stained tRNA loading controls are shown in the lower panels. The RNA expression signals of Tg^BC1_UCU^, Tg^BC1_AUC^ and Tg^BC1^ are similar to BC1 RNA of FVB/N wild-type mouse. As expected, there is no signal in the lane corresponding to the BC1−/− mouse. The expression level of mutated BC1 RNA in Tg^BC1_AG^ mice is around one third, in Tg^BC1_A^ it is almost half and in Tg^BC1_GAU^ mice reduced to a quarter, compared to wild-type animals.

Transgene integration was confirmed by digestion with *Eco*RV followed by Southern blot analysis (Fig. 1). Typically, we estimated one to five transgene copies at different integration sites. We monitored the expression of each mutated RNA by Northern-blot analysis using total mouse brain RNA. BC1 RNA expression levels in Tg^BC1^, Tg^BC1_AUC^ and TG^BC1_UCU^ were comparable to the BC1 RNA expression levels of non-transgenic wild-type mice. When compared to wild-type mice, about two thirds of the expression levels were observed in In Tg^BC1_AG^, about half in Tg^BC1_A^, and only about a quarter in Tg^BC1_GAU^ (Fig. 2).

### Radioactive *in situ* hybridization

**Figure 3:**
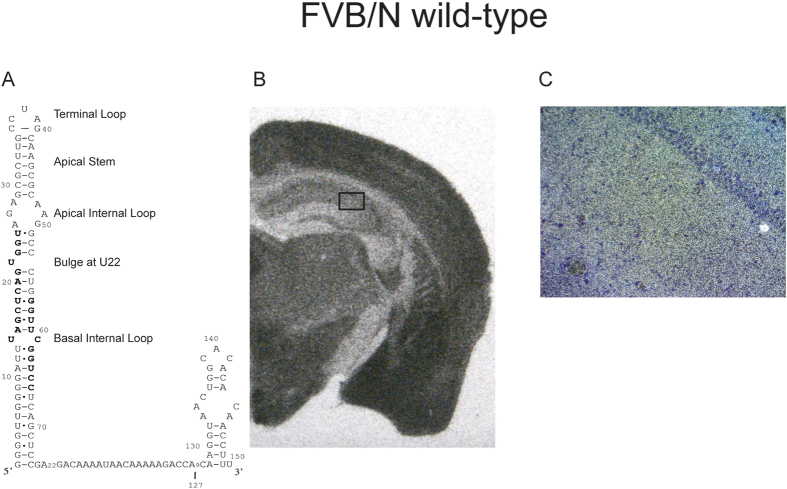
*In situ* hybridization of FVB/N wild-type mouse brain. ISH of BC1 RNA in FVB/N wild-type mouse brain is shown as a control. The structure and the investigated structural motifs of BC1 RNA are shown on the left. Nucleotides corresponding to internal RNA polymerase III promoter elements box A and box B are highlighted in bold letters (**A**). Panel (B) depicts an autoradiography (exposure time: ~20 h). Signal (dark areas) is seen in many areas of the brain in cell bodies as well as, for example, in the dendritic fields of the CA1–CA3 area of the hippocampus. In gyrus dentatus, the signal is weak or absent as expected. Panel (C) is a dark field microscopy picture from the marked area in (**B**) at 200x magnification. The light spots correspond to the silver grains generated by the α-S^35^UTP radiolabeled probe. The signal in the hippocampal CA2 dendritic field indicates that BC1 RNA is transported to the distal extensions of dendrites.

For *in situ* hybridization (ISH) we used wild-type mice and at least three founders for each transgenic mouse line. The probe was *in vitro* transcribed RNA complementary to the 3′ unique part of BC1 RNA (see Materials and Methods). Control brain slices from BC1−/− animals showed no signal either using sense or antisense RNA probes (data not shown). The ISH on wild-type brain slices with the anti-sense probe showed a clear signal in the CA1–CA3 hippocampal areas including the corresponding dendritic fields (Fig. 3C). As expected, transgene-derived RNA variants were prominently expressed in the mouse brain including cortex and hippocampus. As in wild-type mice, no BC1 RNA signal was found in the dentate gyrus. Dark field microscopy depicted the CA2 field in which silver grains appearing as white spots show the hybridization along the entire length of the dendrites in BC1 wild-type mice (Fig. 3C).

### Tg^BC1_AUC^

**Figure 4:**
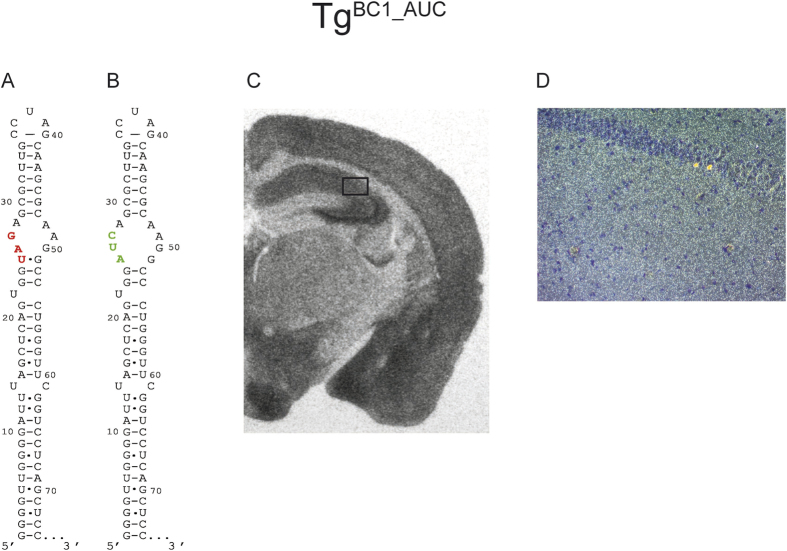
*In situ* hybridization of FVB/N Tg^BC1_AUC^ mouse brain with a mutation in the internal apical loop of BC1 RNA. Panels (A,B) show the structures of the 5′ stem loop of BC1 wild-type RNA and the mutated RNA with the UAG_25–27_ → AUC_25–27_ nucleotide exchange. By introducing the AUC triplet to the RNA, we altered the GA motif. This mutation has no effect on the distal dendritic transport, as can be seen in the autoradiography (exposure time: ~20 h) (Panel C). The signal is visible throughout the dendritic fields of the hippocampus. Panel (D) is a dark field microscopy picture from the marked area in (**C**) at 200x magnification. The light spots correspond to the silver grains generated by the α-S^35^UTP radiolabeled probe. The signal in the hippocampal CA2 dendritic field indicates that this variant of BC1 RNA is transported to the distal extensions of dendrites.

In a previous experiment, it was suggested that the apical internal loop of BC1 RNA possibly forms a non-canonical K-turn structure (GA motif)^18^,^32^. The canonical K-turn motif is characterized by a three-nucleotide bulge, flanked with Watson Crick base pairs at the 5′ and non-canonical GA base pairs at the 3′ end^33^,^34^. It has been proposed that the GA motif may influence dendritic transport^34^ and indeed, *ex vivo*, BC1 RNA devoid of the GA motif could only be detected in the proximal regions of dendrites^18^. Exchange of bases UAG_25–27_ to AUC_25–27_ was expected to eliminate the internal GA motif of BC1 RNA. The nucleotide exchange of AG_26–27_ to UC_26–27_ affected noncanonical GA base pairing within the predicted GA motif (Fig. 4B). The U to A mutation at position 25 could have potentially had an effect on the 3′ flanking stem and was introduced to reproduce the previously reported construct used for testing dendritic transport *ex vivo*^18^. This mutation could have potentially altered the apical internal loop. Additionally, the secondary structure of BC1 RNA predicts a putative pseudoknot structure involving UAG_25–27_ and the terminal loop nucleotides CUA_37–39_ (see below)^35^. Hence, the sequence change to AUC_25–27_ would not only disturb the GA motif, but also this putative pseudoknot formation. *Ex vivo* experiments show a significant reduction of BC1 RNA in dendrites suggesting that this mutation might be important for dendritic targeting^18^. Our ISH experiments with Tg^BC1_AUC^ mice revealed that mutated BC1 RNA distribution in dendrites was not markedly different from wild-type RNA (Fig. 4C,D).

### GA-type targeting motif Tg^BC1_UCU^

**Figure 5:**
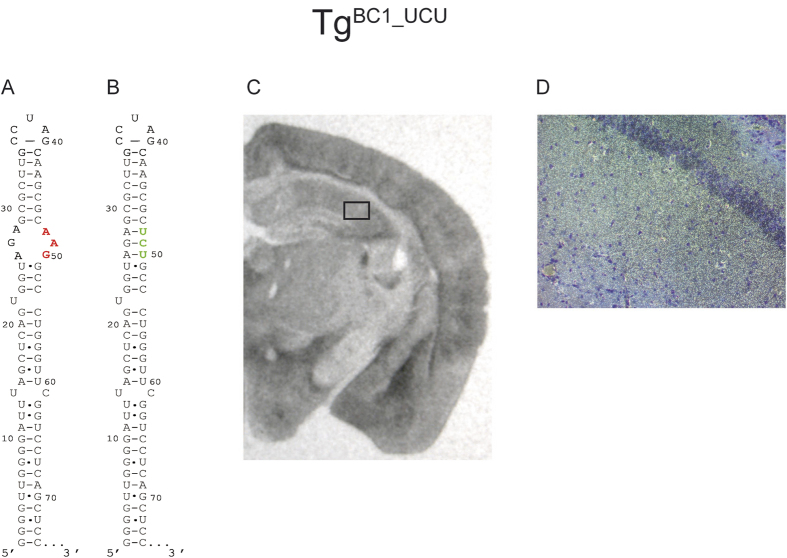
*In situ* hybridization of FVB/N Tg^BC1_UCU^ mouse brain with a mutation in the apical internal loop of BC1 RNA. Wild-type BC1 RNA and the apical internal loop mutation AAG_48–50_ → UCU_48–50_ is shown in panels A and B. This mutation leads to a loss of the apical internal loop by building standard Watson-Crick base pairs. The apical internal loop is predicted to form a GA motif and therefore to play a role in dendritic transport. The autoradiography film (exposure time: ~20 h) (Panel C) shows the expression of mutated BC1 RNA. Panel D is a dark field microscopy picture from the marked area in (**C**) at 200x magnification. The light spots correspond to the silver grains generated by the α-S^35^UTP radiolabeled probe. The signal in the hippocampal CA2 dendritic field indicates that this variant of BC1 RNA is transported to the distal extensions of dendrites.

In addition, we examined a BC1 RNA variant, in which the internal loop nucleotides AAG_48–51_ had been replaced by UCU_48–51_ in order to remove the GA motif by generating standard Watson Crick base pairing, thus extending the canonical RNA alpha helix (Fig. 5B). According to ISH of transgenic mouse brains, the mutated RNA is expressed over the entire brain in regions similar to wild-type animals (Fig. 5C). We were not able to detect a reduction in transport along the dendrites. We could show that this RNA is present in the soma of neurons and, additionally, along the entire lengths of the hippocampal dendrites (Fig. 5D).

### Tg^BC1_GAU^

**Figure 6:**
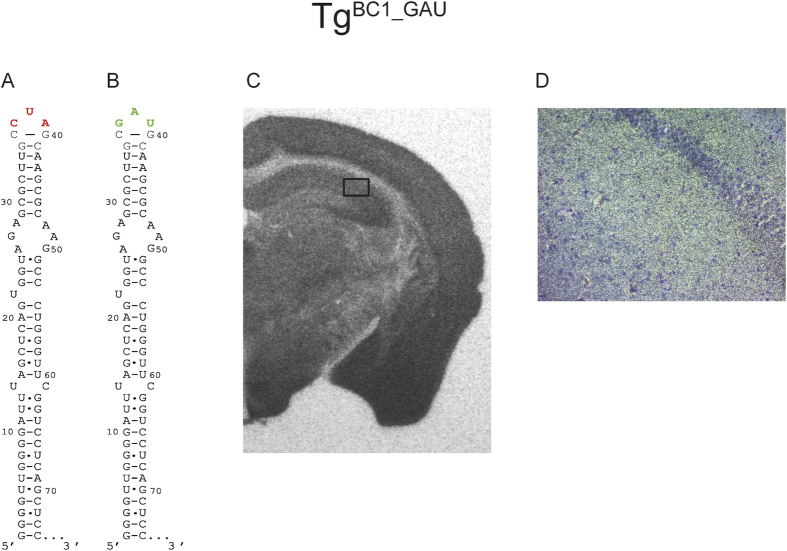
*In situ* hybridization of FVB/N Tg^BC1_GAU^ mouse brain with a mutation in the terminal loop of BC1 RNA. The replacement of bases CUA_37–39_ → GAU_37–39_ in the terminal loop is shown (Panels A,B). We expected this change to interfere with the putative pseudoknot formation between the terminal loop and the UAG_25–27_ nucleotides of the apical internal loop. Also, the interaction of the terminal loop nucleotides with protein(s) necessary for dendritic delivery could be affected. The autoradiography film (exposure time: ~20 h) (Panel C) shows a clear signal corresponding to mutated BC1 RNA in the cell bodies of the hippocampus and the entire dendritic areas CA1–CA3. Panel D is a dark field microscopy picture from the marked area in (**C**) at 200x magnification. The light spots correspond to the silver grains generated by the α-S^35^UTP radiolabeled probe. The signal in the hippocampal CA2 dendritic field indicates that this variant of BC1 RNA is transported to the distal extensions of dendrites.

The 5′ domain of BC1 RNA forms a stem-loop structure with a three-nucleotide terminal loop. As mentioned above, a pseudoknot-structure could be predicted between the terminal loop and UAG_25–27_ nucleotides of the apical internal loop^35^. Although *in vitro* chemical probing experiments did not support the formation of the pseudoknot-structure, RNA folding *in vivo* could differ^20^. In order to test whether the sequence of the single-stranded terminal loop plays a role in dendritic transport, perhaps by interacting with another RNA region (intra- or intermolecularly), or direct binding with protein(s) important for RNA transport, we replaced the terminal loop nucleotides CUA_37–39_ with GAU_37–39_. When ISH was performed on brain slides of Tg^BC1_GAU^ mice we did not observe a reduction in the dendritic transport of mutated BC1 RNA in comparison to wild-type animals (Fig. 6C,D). This is in agreement with *ex vivo* experiments^18^.

### Tg^BC1_A^

**Figure 7:**
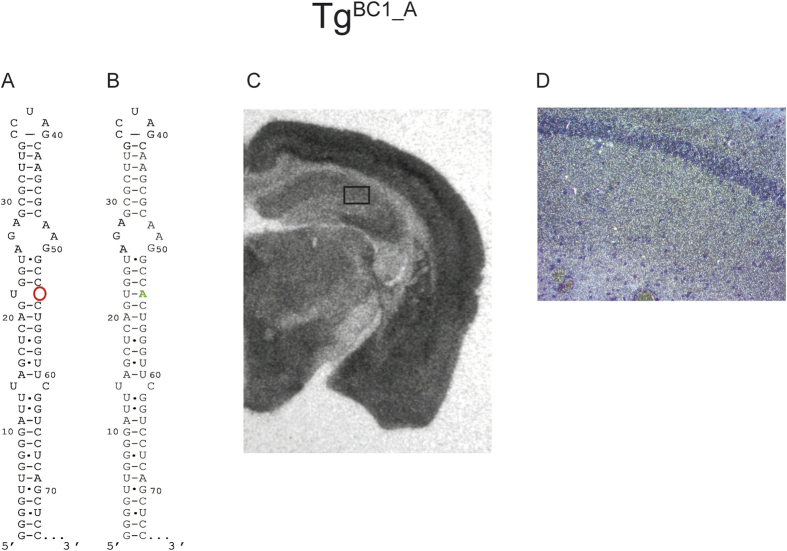
*In situ* hybridization of FVB/N Tg^BC1_A^ mouse brain with a mutation of the U22 bulge of BC1 RNA inserting an A-residue. We inserted an A-residue into BC1 RNA between position 53 and 54 to remove the single nucleotide bulge consisting of the U-residue at position 22. Wild-type BC1 RNA compared to the mutant RNA structures is shown in panels A and B. In contrast to the *ex vivo* RNA microinjection experiments, this variant of BC1 RNA was transported along the entire length of the hippocampal dendrites *in vivo*. The autoradiography film (exposure time: ~20 h) (Panel C) shows a clear signal corresponding to mutated BC1 RNA in the cell bodies of the hippocampus and the entire dendritic areas CA1–CA3. Panel D is a dark field microscopy picture from the marked area in (**C**) at 200x magnification. The light spots correspond to the silver grains generated by the α-S^35^UTP radiolabeled probe. The signal in the hippocampal CA2 dendritic field indicates that this variant of BC1 RNA is transported to the distal extensions of dendrites.

The most drastic effect observed on BC1 transport *ex vivo* is the removal of the bulged U-residue at position 22, resulting in the restriction of RNA to cell bodies^18^. Several attempts to generate a corresponding transgene failed, as the expression levels of the RNA were too low for meaningful analysis by *in situ* hybridization. In order to alternatively remove the bulge structure, we inserted an Adenosine residue after position 54. This generated a U-A base pair, extending the middle stem and thus, also removed the bulge at U_22_. In contrast to *ex vivo* RNA microinjection experiments, this Tg^BC1_A^ RNA variant could clearly be detected in hippocampal dendrites of the respective mice (Fig. 7C,D).

### Tg^BC1_AG^

**Figure 8:**
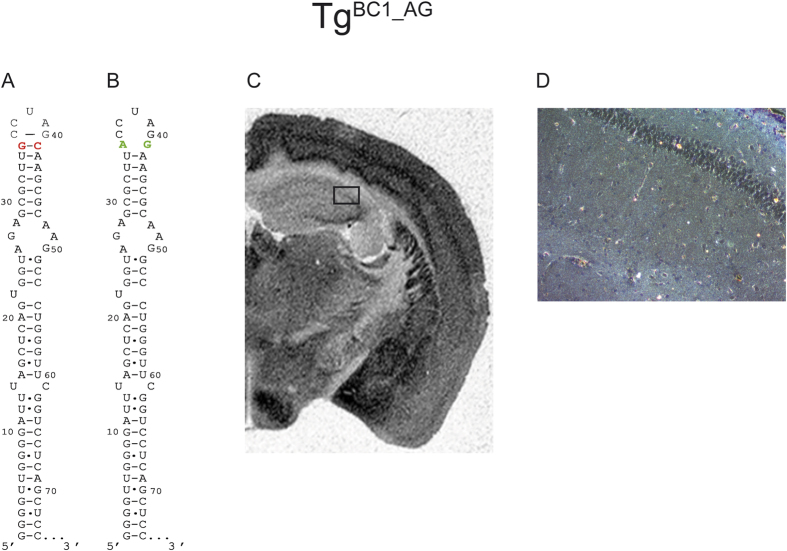
*In situ* hybridization on FVB/N Tg^BC1_AG^ mouse brain with mutation at the terminal loop of BC1 RNA. We generated a mutated BC1 RNA in which we exchanged G_35_C_41_ → A_35_G_41_ (Panel A,B). ID repetitive elements are abundant in rodent genomes. *Ex vivo*, certain ID elements (subclasses ID1 and ID2, but not ID3 or ID4) have been shown to convey dendritic transport to reporter mRNAs. We introduced one of the ID4 hallmark changes into BC1 RNA in order to mimic the ID4 structure at the apical stem. This leads to a loss of transport along the dendritic fields of the hippocampus shown on the autoradiography film (exposure time: ~20 h) (Panel C). Panel D is a dark field microscopy picture from the marked area in (**C**) at 200x magnification. The absence of signal in the hippocampal CA2 dendritic field indicates that this variant of BC1 RNA is not transported into dendrites.

As mentioned in the introduction, rodent genomes contain a large number of ID repetitive elements^22^,^36^. In addition to other positions, members of two ID subfamilies have the following mutations: C_41_ → G_41_ (ID3, ID4) and G_35_ → A_35_ (ID4 only) in the terminal stem loop domain at the penultimate base pair abutting the triloop^20^,^22^. Previous *ex vivo* studies tested two BC1 RNA variants where this penultimate base pair is altered to noncanonical G_35_-G_41_ and A_35_-G_41_, respectively. Both RNA variants were not detected in dendrites under normal conditions but only after potassium polarization^37^. We therefore opted to alter the apical stem in BC1 RNA mimicking the ID4 structure in this region. Hence, we replaced the G_35_-C_41_ Watson Crick base pair with A_35_-G_41_ juxtaposition (Fig. 8B). The ISH experiments on Tg^BC1_AG^ mouse brain slices revealed, in agreement with the aforementioned *ex vivo* experiments, no signal in the dendritic fields of the hippocampus (Fig. 8C). Also, no silver grains were observed in hippocampal dendrites (Fig. 8D). This indicates an inability of dendritic transport of this mutant BC1 RNA *in vivo*.

## Discussion

Much is known regarding the movement of RNA between cellular compartments; this includes the transport of selected mRNAs and various other RNAs, mostly those involved in translation and its regulation from the cell bodies of neurons to their dendritic processes^38^. BC1 RNA is a preferred model to study the relationship between RNA structure and its ability to be transported due to its size (~150 nt), the availability of secondary structure and the absence of posttranscriptional modifications^19^,^20^. Transport competence of BC1 RNA has previously been assayed by microinjection of *in vitro* generated RNA into the cytoplasm of sympathetic neurons in culture^18^,^29^,^39^. These studies suggest the existence of at least two elements within the 5′ stem-loop domain of BC1 RNA; the integrity of which appear to be essential for dendritic transport. The first is a GA motif containing two noncanonical A⋅G pairs^18^,^34^. This structure has been implicated to be important for transport into the distal regions of dendrites^18^. The second essential motif for dendritic transport *ex vivo* is a single base bulge. When the corresponding base (U_22_ in the medial stem) is deleted, RNA transport is completely abolished^18^. A more genuine approach is the investigation of dendritic localization of RNA by expressing various constructs in the brains of transgenic mice (*in vivo*). Previously, there were conflicting *ex* and *in vivo* data concerning the ability of the 5′ stem of BC1 RNA to convey transport competence to chimeric mRNAs. Sequences identical or resembling BC1 RNA were fused to a reporter mRNA (e.g., bicoid RNA from *D. melanogaster*^18^,^33^) or inserted into the 3′ UTR of reporter mRNAs^31^,^39^. The working hypothesis was that dendritic mRNAs might evolve by fortuitous insertion of repetitive ID elements harboring the 5′ stem of BC1 RNA into untranslated regions (UTRs) of an mRNA encoding gene via retroposition. As a consequence, the targeted mRNA might have acquired dendritic transport competence. Injection of reporter mRNA with or without ID element into the cytoplasm of primary culture neurons (e*x vivo*) appeared to confirm this idea^18^,^37^,^40^. However, analogous experiments using transgenic mice could not confirm dendritic location of reporter mRNAs with any variant of the repetitive ID domain of BC1 RNA or even full-length BC1 RNA integrated into the 3′ UTR of a reporter mRNA in either orientation^31^.

Pilot experiments showed that a rat BC1 gene fragment including flanking regulatory regions (~1.4 kb) could be specifically expressed in mouse brains. Although on Northern blots we could differentiate between the transgenic and endogenous RNAs with a short specific antisense oligodeoxynucleotide (not shown), we could not hope to achieve discrimination for *in situ* hybridization due to the high similarity between the rat and mouse RNA (only two nucleotide substitutions). Hence, a modified minigene was constructed replacing 20 A-residues adjacent to the 5′ stem (Fig. 3A) with the triplet CGG. In parallel, we crossed our BC1−/− mice into the FVB/N genetic background facilitating the generation of transgenes by pronuclear injection. This eliminated any concern about possible cross-reactivity with endogenous BC1 RNA, rendering the mini-gene obsolete. We nevertheless tested the minigene along with a rat wild-type BC1 RNA transgenic construct. *In situ* hybridization revealed that both RNAs had a distribution in brain that was indistinguishable from endogenous BC1 RNA in wild-type mice (not shown). A major difference was that the minigene was expressed at significantly lower levels. In general, we observed that transgenes expressed BC1 RNA variants at levels that were comparable to wild-type but some expression was reduced to as low as 25% (see Fig. 2 and Table 1). Importantly, expression levels were never higher than that of endogenous BC1 RNA preempting arguments that overloading the system might lead to aberrant dendritic localization^37^. One factor for expression levels varying from construct to construct might be a correlation with copy numbers and/or loci of integration. Once more, the neuronal expression in specific brain areas of the RNAs apparently did not differ from that of wild-type mice, indicating that all necessary regulatory elements upstream from the RNA coding region are present on the microinjected 1.4 kb fragment. The different expression levels of the various constructs could also be due to alterations that affect the transcription efficiency and/or stability of the variant RNAs. In fact, *in vivo* expression levels sufficient for *in situ* hybridization could not be achieved in a U_22_ deletion construct because the RNA polymerase III A-box promoter consensus sequence was affected (Fig. 3A)^21^. To avoid any consequence on promoter activity, we abrogated the bulge by inserting an A-residue on the opposite side of the stem (between C_53_ and C_54_). While RNA expression was now sufficient, it only reached about 50% of wild-type levels. The two other constructs with lower expression (Tg^BC1_GAU^ and Tg^BC1_AG^) featured nucleotide changes that neither affect box A nor box B (for promoter boxes see Fig. 3A). Despite the lower level of expression for some of the constructs, we could readily identify the presence or absence of dendritic transport of all BC1 RNA variants in our transgenic mouse models.

### GA motif

The GA motif implied in conveying transport competence of RNAs in general and of BC1 RNA in particular was modified in two different ways: First, in Tg^BC1_AUC^, we changed the sequence of the 5′ section of the loop, thus in all likelihood eliminating the GA motif (Fig. 4A). The second (Tg^BC1_UCU^) was to completely replace the apical loop harboring the GA motif by replacing all unpaired as well as noncanonical pairs with bases that form canonical Watson-Crick pairs, thus lengthening the apical stem by three base pairs to altogether fourteen pairs (Fig. 5A). Both variant RNAs were transported into dendrites, albeit not into proximal extensions (up to ~150 μm) *ex vivo*, while transgenic mice expressing these mutant RNAs were virtually indistinguishable from wild-type mice with respect to their location in hippocampal dendritic fields (Figs 4 and 5).

### Triloop sequence

Sequence alteration of the terminal triloop by exchanging all three nucleotides did not impede dendritic transport in either study^18^ ruling out a significance of the triloop sequence as well as the potential formation of a pseudoknot involving the UAG at positions 25–27 and the CUA of the triloop for dendritic transport.

### Bulges

From *ex vivo* data^18^ and our gradually accumulating i*n vivo* data, we did not expect any effect from the removal of the basal internal loop (positions U_14_ - C_61_, see last row of Table 1) and thus, did not generate a corresponding transgene. We rather focused on the *in vivo* analysis of the mutation involving the bulged U-residue at position 22. Removal of this base, thus extending the medial stem to ten bp, virtually abolished dendritic transport *ex vivo* (Table 1)^18^. Surprisingly, *in situ* hybridization signals from our construct when expressed in transgenic mice were well represented in the dendritic fields of the hippocampus (Fig. 7). For the sake of completeness, it should be mentioned that we were unable to obtain useful expression levels of the aforementioned construct in transgenic mice. Therefore, our construct for *in vivo* analysis was slightly different; instead of removing U_22_, we inserted an A-residue between C_53_ and C_54_. Consequently, the resulting medial stem is extended by a U-A pair and is eleven instead of ten bp long, an alteration that is not expected to have a notable impact on the dendritic transport of the RNA variants.

### Triloop structure

The final construct tested, was perhaps the most intriguing. In Fig. 6 we showed that the sequence of the terminal loop is interchangeable and we did not expect much of an effect by replacing the penultimate G-C base pair of the stem closing the triloop into a noncanocical A⋅G juxtaposition. The rationale to chose this variation was based on the following observation: *Ex vivo* experiments using microinjection of *in vitro* transcribed radiolabelled tubulin reporter mRNA with various constructs inserted into the 3′ UTR into the cell bodies of primary neurons found that the 5′ BC1 RNA stem (pos. 1–74) or slight variants thereof, as represented by some ID repetitive SINE elements (subclass ID1 and ID2) impart dendritic transport behavior on the reporter mRNA^37^. In contrast, subclass ID3 and ID4 (featuring G⋅G and A⋅G juxtaposition at the aforementioned base pair), respectively, failed to do so under normal conditions. The authors claim that dendritic transport is restored by increased KCl concentration and suggested a model of activity dependent RNA transport in neurons^37^. In any event, although our previous study did not reveal dendritic transport with BC1 RNA or various ID elements inserted into the 3′ UTR of EGFP reporter mRNA^31^, we reproduced the corresponding variation in the penultimate base pair abutting the triloop to a transgene. Interestingly, in mice, perhaps with the exception of some transport over short distances into proximal parts of neuronal processes, there was a complete absence of *in situ* hybridization signal in dendritic fields of the hippocampus. This is consistent with *ex vivo* experiments using the corresponding unfused BC1 RNA (A_35_⋅G_41_)^37^. Among all possible explanations, we would like to offer the following: Exchange of a G-C pair with an A⋅G juxtaposition potentially changes the structure important for triloop formation. Hence, the triloop structure and not the corresponding unpaired loop bases is an important determinant for dendritic transport of BC1 RNA.

From our *in vivo* study of dendritic transport, it appears that the two structural elements implied in the past, namely the apical GA motif and the single bulge, which have been shown *ex vivo* to alter the intracellular localization of BC1 RNA^18^,^32^, do not markedly diminish RNA dendritic transport in hippocampal neurons of transgenic mice. We are aware of the possibility that this might differ in other neuronal cell-types or as a consequence of experimental treatment^37^. Here, we identified the structure supporting the triloop as a strong candidate for conferring dendritic transportability to BC1 RNA. As shown with the transgene expressing the Tg^BC1_GAU^ variant, the actual three-base sequence of the loop is apparently irrelevant. This is further supported by phylogenetic information: in squirrel (*Sciurus carolinensis*) the unpaired loop sequence is altered from CUA → CUU^41^ (and unpublished data).

Despite the fact that transgenic mouse experiments are time consuming, animal welfare restricted and expensive, we felt that *in vivo* analysis of different BC1 RNA structures influencing dendritic targeting was an important contribution to our understanding of structure/function relationships for RNA transport.

## Materials and Methods

### Generation of the rat BC1 RNA transgenic mice

For transgenic mouse generation we used the Sleeping Beauty transposase system in vector pT2BH^42^. The *Sac*I-*Bam*HI 1.4 kb DNA fragment containing the wild-type rat BC1 RNA gene together with 5′ and 3′ flanking regions from the pBC1 plasmid^21^ was cloned into *Eco*RV-*Bgl*II sites of the pT2BH. The resulting plasmid pT2BH_BC1 was used for further mutagenesis. Transgenic mice were generated through regular pronuclei injection in FVB/N BC1−/− embryos as described previously^31^,^43^. The Tg^BC1_AUC^ and the Tg^BC1_GAU^ mutations were generated in the pT2BH_BC1 using pairs of oligonucleotides UAG_AUC_DIR/UAG_AUC_REV and CUA_GAU_DIR/CUA_GAU_REV respectively (Table 2). The Tg^BC1_AG^ mutation was generated in pBC1 directly with the aid of oligonucleotides GC-AGfsp/GC-AGrsp (Table 2). Mutagenesis was performed using the PCR approach utilizing Taq and Fusion thermostable enzymes with consecutive sequencing verification. The Tg^BC1_UCU^ and Tg^BC1_A^ constructs were synthesized and cloned into the pT2BH vector commercially (Blue Heron, Bothell, WA, USA).

### DNA isolation and Southern blot analysis

We performed tail biopsies from three-week-old transgenic mice. The DNA was isolated via the phenol/chloroform extraction method. The DNA was digested with *Eco*RV restriction enzyme (Fermentas) and separated on a 0.8% agarose gel. Following electrophoresis, the DNA was transferred by capillary blotting onto a GeneScreen hybridization membrane (Perkin Elmer). BC1 transgenic probe 800 bp DNA fragment downstream from an *Eco*RV site located in the 3′ flank of the BC1 RNA gene was labeled with [γ-^32^P]-dCTP (PerkinElmer) and high prime DNA labeling Kit (Roche).

### RNA isolation and Northern blot analysis

Total brain RNA was isolated using the Trizol reagent according to the manufacturer’s manual. Ten μg of total RNA was separated on 8% (w/v) polyacrylamide [29:1 acrylamide/bisacrylamide], 7M urea gel and electro-transferred onto a positively charged nylon membrane (Roche). Hybridization probe (BC1 unique deoxyoligonucleotide, 50 pmol, Table 2) was radioactively labeled using [γ-^32^P]-ATP (PerkinElmer) and T4 polynucleotide kinase (Fermentas) according to the manufacturer’s instructions. Northern blot analysis was performed as previously described^44^. To estimate expression levels of wild-type BC1 RNA and variants, we used the Advanced Data Image Analyzer software (AIDA Vers4.26.038, Raytest). Densiometry of the autoradiography film revealed expression of the mutated BC1 RNAs ranging from around one quarter (~24%) to almost equal (~90%) compared to endogenous wild-type mouse RNA (Table 1).

### Radioactive *in situ* hybridization

Mice (aged 8 to 24 weeks) were sacrificed with CO_2_ and subsequently perfused with 1x PBS media and 4% paraformaldehyde. Brains were sectioned with a vibratome (Leica) into 30 μm coronal slices. Hybridization was performed as previously described^45^. RNA probes were generated from pMK1 plasmid as template^24^. DNA was linearized with *Sac*I and transcribed by T3 RNA polymerase for the sense probe. The antisense probe was generated by T7 RNA polymerase from pMK1 linearized with *Asp*718I. In both cases, *in vitro* transcription was performed in the presence of α-S^35^UTP. RNA polymerases and restriction enzymes were purchased from Thermo Scientific. pMK1 sense probe: 5′-gggaacaaaagcuggguaccAAAAAAGACAAAAUAACAAAAAGACCAAAAAAAAACAAGGUAACUGGCACACACAACCUUUUg-3′; pMK1 antisense probe: 5′-gggcgaauuggagcucAAAAGGUUGUGUGUGCCAGUUACCUUGUUUUUUUUUGGUCUUUUUGUUAUUUUGUCUUUUUUg-3′. Lower case letters represent transcribed vector sequences.

For hybridization, we used RNA with 4.5 × 10^6^ cpm and incubated in hybridization buffer at 50 °C overnight.

The slices were washed once with 2x SSC and deionized formamide at a ratio of 1:1 at 50 °C and once with 2x SSC at the same temperature. Subsequently, we treated the slices with 30 μg/ml RNase A at 40 °C and washed again in 5 liters 2x SSC at 50 °C for 3 h and continued washing in the same buffer at room temperature overnight.

The slices were mounted onto Superfrost Gold Plus (Menzel) microscopy slides, dried with increasing concentrations of 50%, 70% and 90% ethanol and exposed to autoradiography films (Kodak Biomax MR) overnight.

After developing the films the slides were dipped into autoradiography emulsion (Kodak NTB) as previously described^24^ and kept at +4 °C for 10 days. To develop the slides, we used developer D-19 and Rapid Fixer (Kodak). The brain slices were stained with cresyl violet using an autostainer (Leica). Dark field pictures were taken with an Axio imager microscope (Zeiss) at 200x magnification.

### Mice

All mouse procedures were performed in compliance with the guidelines for the welfare of experimental animals issued by the Federal Government of Germany and approved by the State Agency for Nature, Environment and Consumer Protection North Rhine-Westphalia (Landesamt für Natur, Umwelt und Verbraucherschutz Nordrhein Westfalen)^45^. Animals were kept in specific pathogen-free animal facilities. All breeding was conducted in a controlled (21 °C, 30–50% humidity) room with a 12:12 hour light-dark cycle. Mice were housed under non-enriched, standard conditions in individually ventilated (36 (l) × 20 (w) × 20 (h) cm) cages with up to five littermates. Pups were weaned 19–23 days after birth and females were kept separately from males^8^.

## Additional Information

**How to cite this article**: Robeck, T. *et al.* BC1 RNA motifs required for dendritic transport *in vivo*. *Sci. Rep.* **6**, 28300; doi: 10.1038/srep28300 (2016).

## Figures and Tables

**Table 1:** Comparison of *ex vivo* data (microinjection of *in vitro* transcribed radiolabelled RNA into cell bodies of primary neurons^18^) with *in vivo* results (transgenic mice expressing BC1 RNA variants in a strain where the endogenous gene had been deleted^11^).

Designation here and figure #	RNA abundance in % of wt	Designation refs 18 and 37	*in vivo*	*ex vivo*	structure affected
wt BC1	~91%	wt	+++	+++	
BC1_AUC (4)	~90%	IL-A:AUC	+++	++	apical internal loop, change
BC1_UCU (5)	~89%	IL-A:WC	+++	++	apical internal loop, close (bp)
BC1_GAU (6)	~25%	TL-GAU	+++	+++	terminal triloop
BC1_A (7)	~50%	B22: U22 Delet	++	(+)	bulge, U22*
BC1_AG (8)	~71%	BC1 RNA A35⋅G41	(+)	(+)	penultimate base-pair below triloop**
n.d.		IL-B:WC		+++	basal internal loop

Symbols for semi-quantitative assessment of dendritic transport are as follows: +++, transport beyond 200 μm from soma; ++ transport up to 150 μm from soma, (+), none or perhaps in proximal dendrites. The *ex vivo* data are taken from^18^. Assessment of location distance and quantity are more limited by *in situ* hybridization data. *It should be noted that the constructs used *in vivo* and *in vitro* concerning the bulged U_22_. For the former, an additional A residue was inserted opposite from U_22_ after position 53, and for the latter U_22_ simply had been removed.

**Table 2:** Oligonucleotides used in this Study.

Oligo name	Sequence
GC-AGfsp	5′-CGCTTCCTAGGTAAGCGCTCTACCACTGAGC-3′
GC-AGrsp	5′-CGCTTACCTAGGAAGCGCAAGGCCCTGGGTTCG-3′
UAG_AUC_DIR	5′-GGATCAGCGCTTGCCTAGCAAGCGCAAGGCCCTGGGTTCGGTCCTCAGCTCC-3′
UAG_AUC_REV	5′-CCTTGCGCTTGCTAGGCAAGCGCTGATCCACTGAGCTAAATCCCCAACCCCGA-3′
CUA_GAU_DIR	5′-GTAGAGCGCTTGCGATGCAAGCGCAAGGCCCTGGGTTCGGTCCTCAGCTCC-3′
CUA_GAU_REV	5′-CTTGCGCTTGCATCGCAAGCGCTCTACCACTGAGCTAAATCCCCAACCCCGA-3′
BC1 unique	5′-AAAAGGTTGTGTGTGCCAGTTACCTTGTTTTT-3′
